# Asynchronous Monitoring-Induced Deviation in Hydraulic Support Peak Estimation and Its Sensitivity to Synchronization and Sampling Resolution: A Case Study of a Solid Backfilling Working Face

**DOI:** 10.3390/s26175660

**Published:** 2026-09-06

**Authors:** Tingcheng Zong, Qiang Zhang, Kang Yang, Zishan Jin, Pengfei Cui, Jinhong Song, Ruiyi Zhang, Xianqi Ning

**Affiliations:** 1 School of Mines, China University of Mining & Technology, Xuzhou 221116, China; tb24020047a41ky@cumt.edu.cn (T.Z.); tb22020033a41ld@cumt.edu.cn (K.Y.); tb25020022a41@cumt.edu.cn (Z.J.); ts21020005a31tm@cumt.edu.cn (P.C.); ts25020192p31@cumt.edu.cn (J.S.); ts25020090a31@cumt.edu.cn (R.Z.); ts25020182p31@cumt.edu.cn (X.N.); 2 Key Laboratory of Deep Coal Resource Mining, Ministry of Education, China University of Mining and Technology, Xuzhou 221116, China; 3 School of Energy, China University of Mining and Technology, Xuzhou 221116, China

**Keywords:** pressure sensor monitoring, hydraulic support, asynchronous sampling, peak definition, sampling resolution, solid backfilling

## Abstract

Hydraulic-support pressure monitoring is widely used for mining-pressure interpretation and roof-weighting event statistics, but asynchronous records, incomplete spatial coverage and low-frequency instantaneous sampling can change the engineering quantity represented by a cycle peak. This study audits a continuous three-month pressure dataset from a large-height solid backfilling working face. The stored asynchronous available-section peak was reconstructed; synchronized face-mean, maximum section-mean and maximum support peaks were calculated on an exact 3 min grid; and multi-phase down-sampling was evaluated at 15, 30 and 60 min relative to the field high-frequency reference. The stored peak was determined from only one valid monitoring section in 240 of 255 cycles and exceeded the synchronized average pressure peak of the three monitored sections by 19.7 MPa on average, with weak overlap between their statistical roof-weighting labels. Regional and support-level synchronized indicators additionally identified local high-resistance responses that were not classified by the face-mean indicator under their respective thresholds. At 60 min, the mean retained-event rate across sampling phases was 0.484 relative to the field reference. Filling-advance ledgers and roadway-stress records showed exploratory associations with a restricted subset of joint events after correction for multiple testing. The results establish reporting requirements for peak definition, synchronization completeness, sampling resolution and engineering use before support-pressure peaks are used for event statistics or prediction.

## 1. Introduction

Hydraulic-support pressure is one of the most direct and continuously available measurements for mining-pressure analysis in longwall coal mining. Studies based on hydraulic-support load records have used these data for support-load analysis, load prediction and mining-pressure monitoring in backfilling working faces [1,2]. Recent support-resistance and surrounding-rock-pressure studies further show that hydraulic-support monitoring data can be used to model spatiotemporal pressure responses in working faces [3,4]. Therefore, before pressure data are used for prediction or event statistics, the monitored pressure variable itself must be clearly defined.

In practical multi-support monitoring datasets, records from different supports or monitoring sections are not always perfectly synchronized. Data export procedures, communication delay, temporary sensor dropout and storage strategies can cause a raw timestamp to contain valid records from only part of the working face. If the mean pressure of the available sections is calculated at each raw timestamp and the maximum value is then selected within a production cycle, the resulting peak represents an asynchronous available-section peak. If the objective is to describe the synchronized average pressure response of the three monitored sections, the monitored sections should first be aligned onto a common time grid, and the face mean should then be maximized within the cycle. These two quantities have different engineering meanings and should not share the same threshold or event interpretation.

Sampling resolution is another source of uncertainty in support-pressure analysis. Longer sampling intervals may miss short-duration cycle peaks, change statistical roof-weighting labels and alter the inferred dominant pressure-response section along the face. This issue is especially relevant when low-frequency instantaneous records are used instead of window maxima or event-triggered records. Therefore, the sampling interval, peak-preservation rule and synchronization completeness should be reported together when support-pressure data are used for mining-pressure event statistics.

Previous studies have advanced mining-pressure and hydraulic-support-pressure prediction from several directions. Early predictive studies modelled first weighting, roof pressure and hydraulic-support load using genetic algorithm–backpropagation (GA-BP), grey neural networks and time-series data modelling [1,5,6]. Data-model-driven roof-disaster prediction and multiple-regression-based mining-pressure prediction using hydraulic-support monitoring datasets have also been reported [7,8]. Graph structure learning, long short-term memory (LSTM) with transfer learning and adaptive graph convolutional recurrent network (AGCRN) models have been used for underground-pressure forecasting, roof-rock stress prediction and working-face pressure prediction [9,10,11]. Other studies have applied the Nesterov-accelerated adaptive moment estimation optimizer with LSTM (Nadam-LSTM), clustering reconstruction and deep-learning classification to working-face pressure prediction, abnormal mining-pressure analysis and regional roof-pressure evaluation [12,13,14]. Deep spatiotemporal sequence models, temporal–spatial correlation frameworks and data-driven pressure-exertion prediction methods have further been developed for support-resistance or surrounding-rock-pressure prediction [3,4,15]. More recent studies have introduced multilevel temporal feature integration, LSTM–Transformer models and gated recurrent unit with an attention mechanism (GRU-AM) for hydraulic-support pressure prediction or abnormal pressure-event recognition [16,17,18]. Multi-head attention and temporal feature learning have also been integrated for hydraulic-support pressure prediction in a coal longwall face [19]. These studies confirm the value of hydraulic-support monitoring data for mining-pressure prediction. However, most prediction-oriented studies assume that the input peak values and event labels have already been reliably defined. If the original multi-support records are asynchronous, spatially incomplete or stored at low temporal resolution, the peak variable may have changed its engineering meaning before entering the prediction model.

Hydraulic-support working resistance is affected by mining height, support parameters, and the surrounding geological and mining conditions; geological–geomechanical characterization is therefore required when interpreting measured mechanical responses or transferring pressure thresholds between working faces. Studies of large-mining-height, parameter-varying, and deep coal-pillar working faces illustrate these controls [20,21,22]. More broadly, integrated geological–geomechanical characterization has been used to relate stratigraphic architecture and geomaterial properties to mechanical response in complex sedimentary settings [23]. These considerations indicate that the pressure variable used in analysis should be consistent with the engineering target. A synchronized face-mean peak is suitable for describing the average pressure response of the three monitored sections, whereas a maximum section-mean peak or a maximum single-support peak is more appropriate for regional high-resistance checks or support-level safety checks.

For solid backfilling working faces, support-pressure interpretation should also consider the backfilling context. Mining-pressure monitoring and strata-behaviour studies in fully mechanized solid backfilling faces have examined the relationship between support pressure and strata behaviour under backfilling conditions [2,24]. Filling ratio and shield supporting pressure have been analysed for overburden movement control in compacted backfilling mining [25], and roof stability has been studied under nonlinear compaction of a solid backfill body [26]. These studies show that backfilling conditions are relevant to support-pressure response. Nevertheless, daily or cycle-allocated advance and backfill ledgers are production proxy variables; they are not direct measurements of real-time filling ratio, equivalent mining height or roof movement. In this study, backfill-advance information and roadway-stress data are therefore used only as supplementary event-screening evidence after the pressure peak definitions have been audited.

This paper focuses on a monitoring-definition problem that precedes prediction modelling. Using the Mine 01 large-height solid backfilling working face as a field case, four questions are examined: (1) whether an asynchronous available-section peak can represent a synchronized face-mean pressure peak calculated from the three monitored sections; (2) how synchronized face-mean, synchronized maximum section-mean and synchronized maximum support peaks should be interpreted; (3) how peak underestimation and statistical roof-weighting event retention change when the field high-frequency reference (median raw interval approximately 2.92 min) is down-sampled to 15, 30 and 60 min; and (4) whether peak definition and sampling interval change the dominant pressure-response section and spatial peak range. The contribution is a reproducible sensor-data audit framework for aligning pressure-peak definitions, synchronization rules, sampling resolution and engineering interpretation in asynchronous multi-support monitoring.

## 2. Materials and Methods

**Figure 1:**
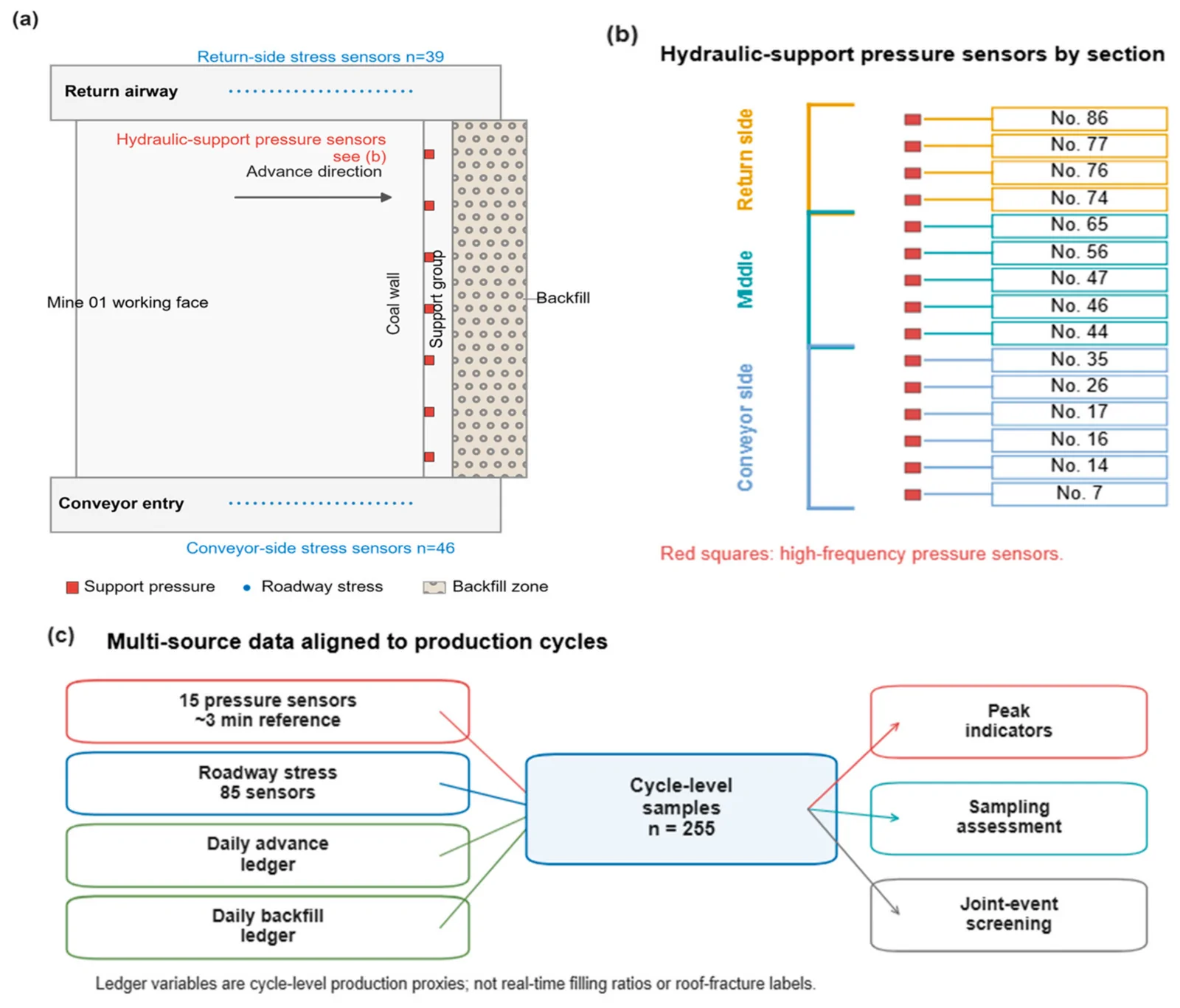
Working-face layout and multi-source sensor monitoring locations in the Mine 01 working face. (**a**) Layout of the working face, conveyor entry, return-airway entry, advance direction, hydraulic-support group and backfill body. (**b**) Locations of 15 hydraulic-support pressure monitoring points and three monitoring sections. The conveyor-side section includes supports 7, 14, 16, 17, 26 and 35; the middle section includes supports 44, 46, 47, 56 and 65; the return-airway-side section includes supports 74, 76, 77 and 86. (**c**) Alignment of roadway stress, support pressure, daily advance ledger and daily filling ledger onto production-cycle samples.

The working-face layout, support-pressure sensors, roadway-stress sensors and production ledgers used in this study are shown in Figure 1.

**Figure 2:**
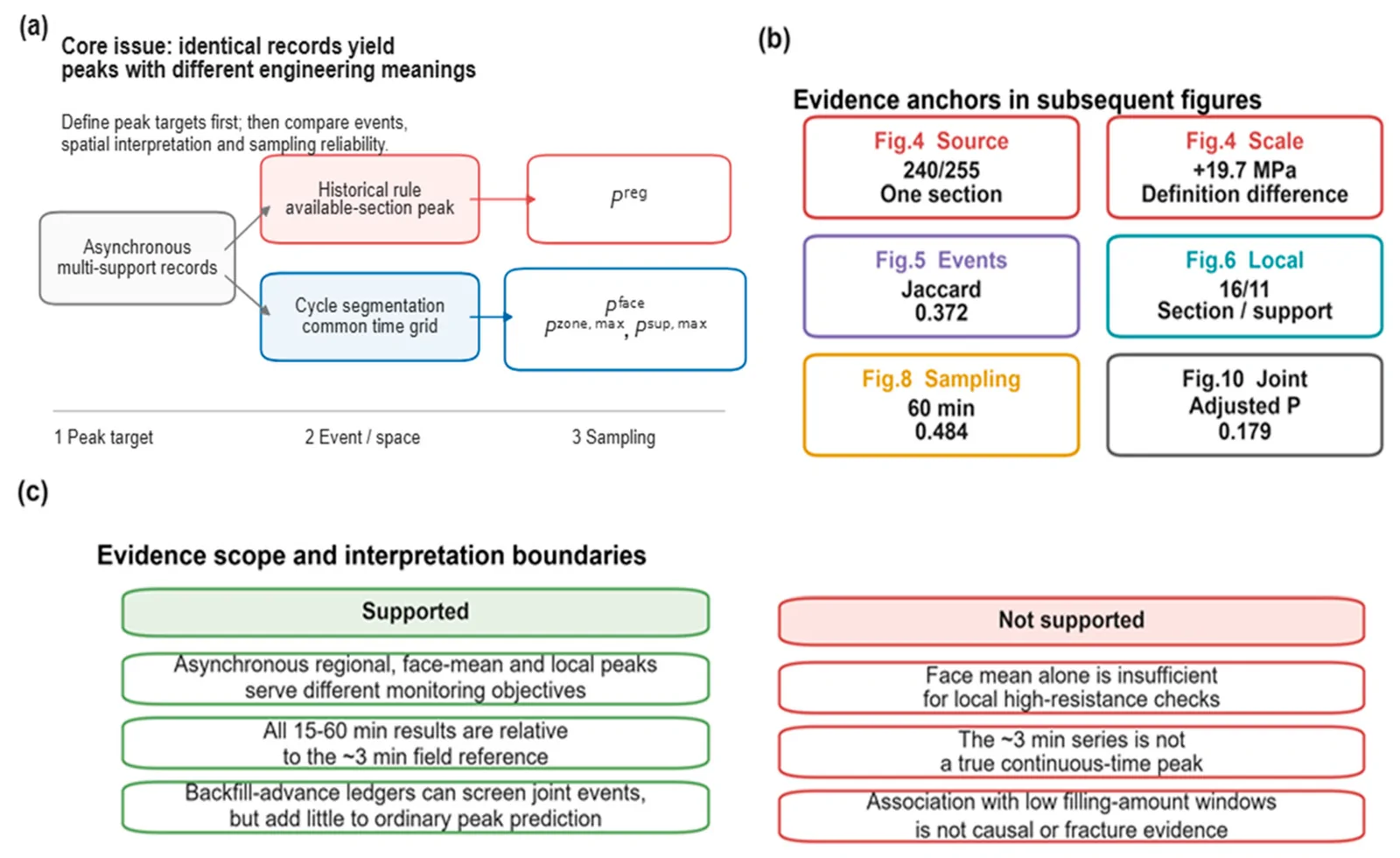
Study workflow and evidence boundary. (**a**) Asynchronous regional peaks and synchronized peak indicators have different engineering meanings in multi-support monitoring. (**b**) Main evidence anchors, including historical peak-source reconstruction, peak-scale shift, statistical label change, attenuation of local high-resistance responses by spatial averaging, sampling-retention loss and backfill-stress joint-event screening. (**c**) The supported interpretation range and the boundaries that should not be exceeded. The colored backgrounds are used only for visual distinction between the three subpanels (**a**–**c**) and do not carry additional semantic information. Panel (**b**) indicates the locations of subsequent evidence-anchor figures (Figures 4–6, 8 and 10).

The evidence chain is organized in three layers: pressure-peak definition, parameter sensitivity and supplementary backfill-stress joint-event screening. The overall workflow and evidence boundary are shown in Figure 2.

### 2.1. Field Background and Multi-Source Monitoring Data

The Mine 01 working face is a large-height solid backfilling working face equipped with multiple hydraulic-support pressure monitoring points. The main analysis used high-frequency monitoring records from October to December 2025. After invalid timestamps, support identifiers and pressure values were removed, the dataset contained 589,011 valid support-pressure records from 15 fixed monitored supports and three monitoring sections. According to within-month production-cycle boundaries, 255 high-frequency cycles were constructed as the analysis units. The October–December interval was selected because it provided a continuous three-month production sequence with high-frequency pressure records, identifiable within-month cycle boundaries, and contemporaneous advance, filling and roadway-stress records. It is treated as a case-study interval rather than as evidence of an annual pattern.

The 15 monitored supports were divided into three spatial sections: conveyor side, middle and return-airway side. At a given timestamp, the pressure value of each section was calculated from valid monitored supports in that section. Because the three sections contained different numbers of monitored supports (4–6 supports), the equal-section face mean represents the average response of the three areas rather than a support-number-weighted average across the monitored supports. A support-number-weighted mean was also calculated as a sensitivity check. The raw sampling interval had a median value of approximately 2.92 min; this series is the field-available high-frequency reference, not the true continuous-time pressure peak. All down-sampling losses reported in this paper are relative to this field reference. The target intervals of 15, 30 and 60 min are exact 5-, 10- and 20-step multiples of the 3 min synchronization grid and cover progressively coarser monitoring resolutions; traversing all feasible phase offsets then evaluates sensitivity to the sampling origin.

Supplementary analyses also used daily advance ledgers, daily filling ledgers and roadway coal-stress records from the same period. Filling amount and advance distance were used to estimate cycle-scale filling amount per unit advance and windows comprising cycles with a relatively low filling amount per unit advance. These are production-ledger proxy variables and are not equivalent to real-time filling ratio. The roadway-stress dataset contained 57,660 records from 85 monitoring points, including 46 conveyor-entry points and 39 return-airway-entry points. It provided stress-response evidence independent of support-pressure records.

**Figure 3:**
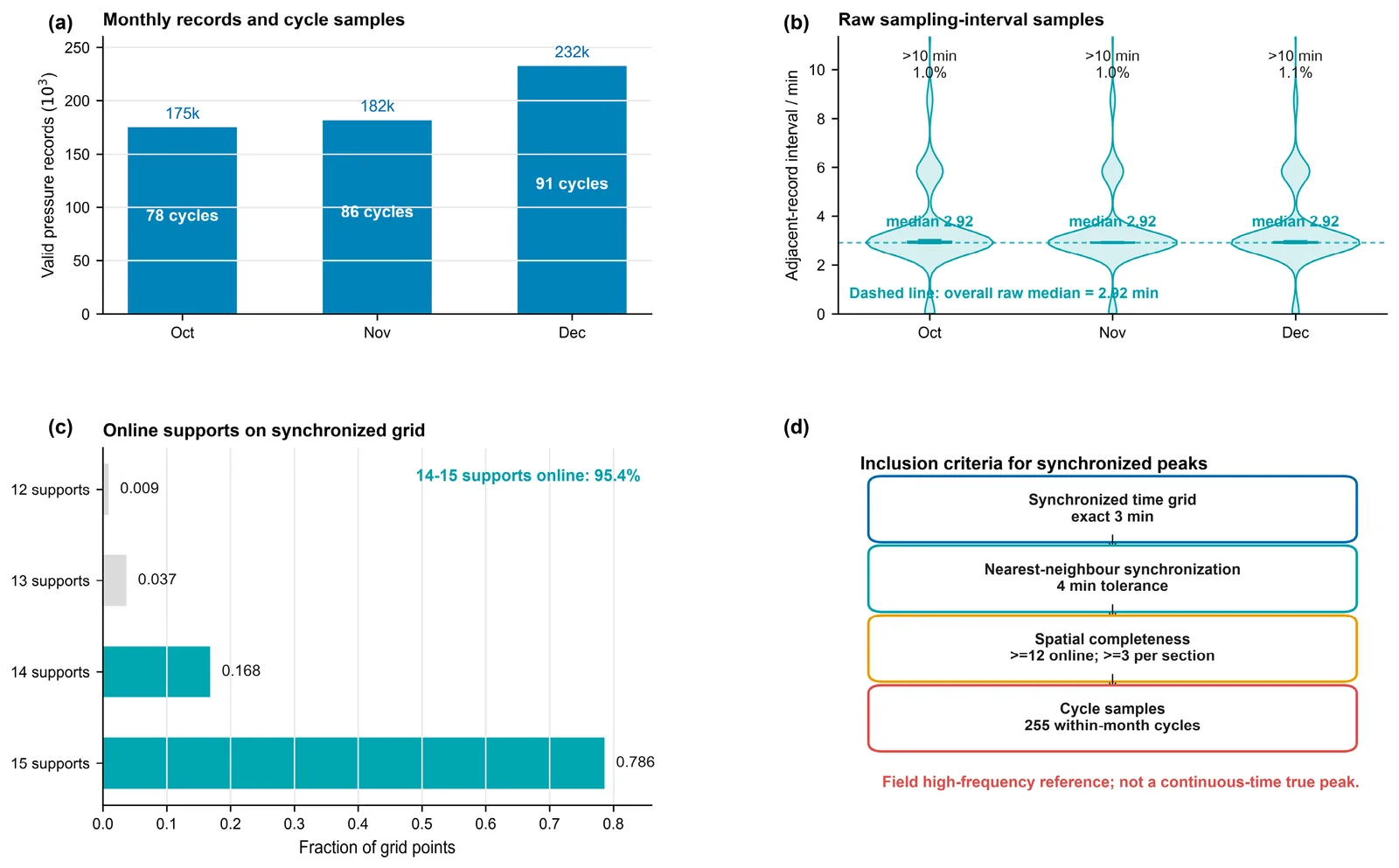
Data availability and synchronization-analysis boundary. (**a**) Valid pressure-record numbers and within-month cycle numbers from October to December 2025. (**b**) Monthly sampled distributions of adjacent raw-record intervals; approximately 2.92 min is the median interval of the field high-frequency reference. (**c**) Online-support counts using a 4 min matching tolerance on the exact 3 min grid. (**d**) Inclusion criteria for synchronized peak analysis, including unified time grid, nearest-neighbour matching, spatial completeness and within-month cycles.

The data scale, sampling reference and synchronization boundary are summarized in Figure 3 and Table 1. The exact 3 min grid is a constructed synchronization reference; the approximately 2.92 min value describes the median interval of the raw field records.

### 2.2. Asynchronous Regional Peak and Synchronized Peak Indicators

For cycle (c), let (t) denote a raw timestamp, (*R_t_*) denote the set of monitoring sections with valid records at (t), and (${\bar{P}}_{r}(t)$) denote the mean pressure of section (r). The historical aggregated peak was reconstructed using Equation (1):

(1) $$P_{c}^{\mathrm{reg}}\;=\;\max_{t\in c}\left[\frac{1}{\left|R_{t}\right|}\sum_{r\in R_{t}}{\bar{P}}_{r}(t)\right]$$

When (|*R_t_*| = 1), a single section mean directly determines the cycle peak. This variable is therefore referred to as the asynchronous available-section mean peak, abbreviated as the asynchronous regional peak.

To calculate the synchronized face-mean peak as the equal-section average of the three monitored sections, the 15 support records were aligned onto a unified exact 3 min time grid using nearest-neighbour matching. Only grid points satisfying the spatial-completeness requirement were used: a 4 min matching tolerance, at least 12 online supports and at least 3 valid supports in each section. The 4 min tolerance was prespecified as an upper bound that covers the observed field-reference timing variability while avoiding unconstrained matching across adjacent cycles. For accepted matches, the median absolute offset from the synchronized grid was 0.883 min, the 95th percentile was 2.700 min, the 99th percentile was 3.583 min, and the maximum was 4.000 min. The synchronized face-mean peak was then defined by Equation (2):

(2) $$P_{c}^{\mathrm{face}}\;=\;\max_{t\in c}\left[\frac{1}{3}\sum_{r=1}^{3}{\bar{P}}_{r}(t)\right]$$

This variable describes the synchronized average pressure response of the three monitored sections and is used as a proxy for the monitored-face load background. It should not be used alone as a local support-safety indicator. To keep local high-resistance checks within the same synchronized grid, Equations (3)–(5) were calculated:

(3) $$P_{c}^{\mathrm{wface}}=\max_{t\in c}\left[\frac{1}{\left|J_{t}\right|}\sum_{j\in J_{t}}P_{j}(t)\right]$$

(4) $$P_{c}^{\mathrm{zone},\max}=\max_{t\in c}\max_{r=1,2,3}{\bar{P}}_{r}(t)$$

(5) $$P_{c}^{\sup,\max}=\max_{t\in c}\max_{j\in J_{t}}P_{j}(t)$$

where (*J_t_*) is the set of online monitored supports at synchronized time (t). ($P^{\mathrm{wface}}$) is the support-number-weighted synchronized mean, ($P^{\mathrm{zone},\max}$) is the synchronized maximum section-mean peak, and ($P^{\sup,\max}$) is the synchronized maximum support peak. These variables respectively serve as a weighted-mean sensitivity check, a regional high-resistance check and a support-level high-resistance check.

**Figure 4:**
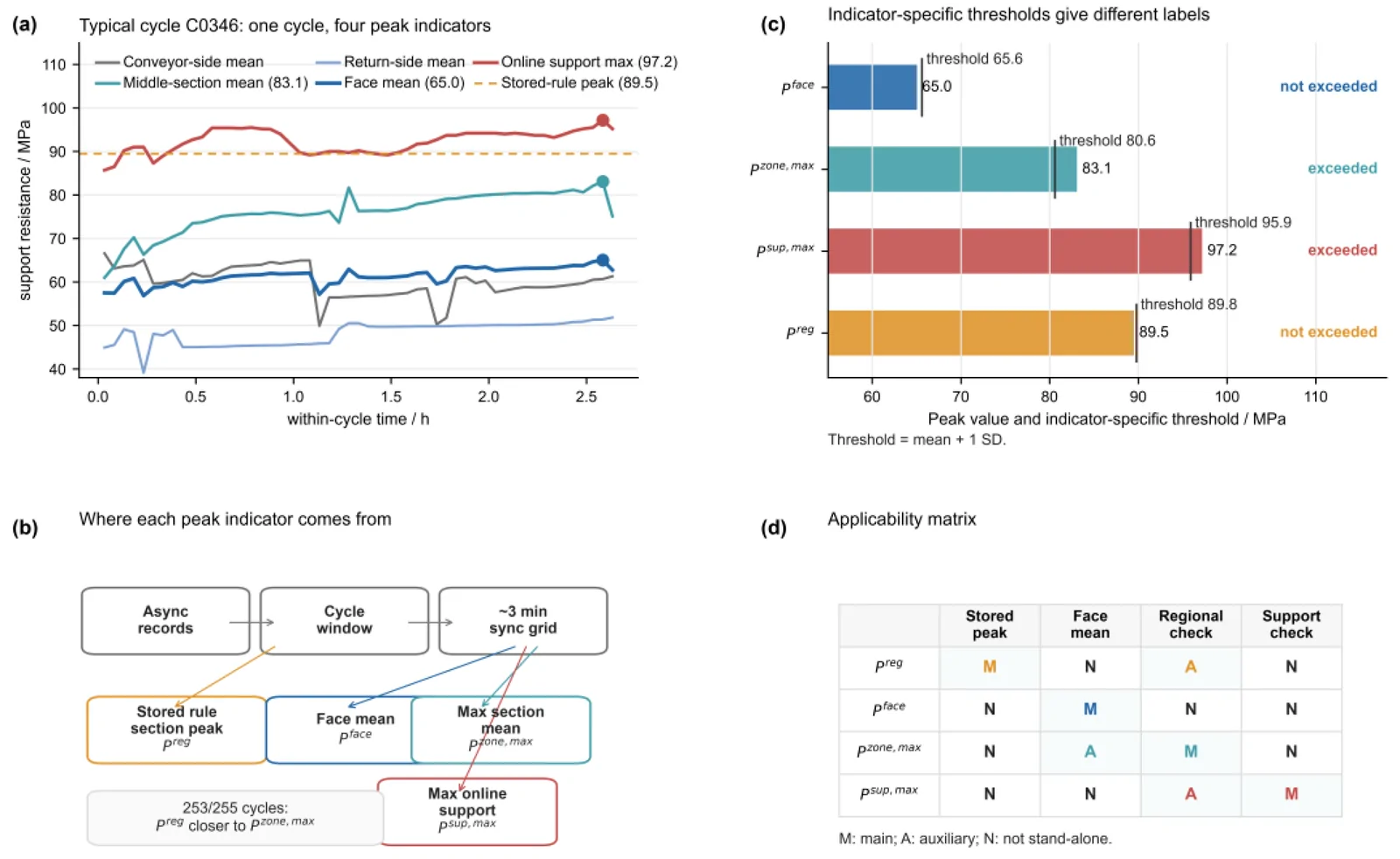
Cycle-level pressure-peak indicator definitions. (**a**) A typical cycle showing differences among the asynchronous regional peak, synchronized face-mean peak, synchronized maximum section-mean peak and synchronized maximum support peak. (**b**) Transformation from raw asynchronous records to cycle boundaries, synchronized time grids and four peak indicators. (**c**) Different statistical roof-weighting judgements obtained using indicator-specific thresholds. (**d**) Recommended use and unsuitable use of each peak indicator. In panel (**a**), the orange dashed horizontal line marks the stored historical regional peak, and the coloured circular markers identify the synchronized within-cycle maxima. In panel (**c**), black vertical lines denote indicator-specific mean + 1 SD thresholds calculated from the 255-cycle sample. The stored historical regional peak reproduces the recorded algorithmic variable; the synchronized face-mean peak describes the average pressure response of the three monitored sections; the synchronized maximum section-mean peak supports regional high-resistance checks; and the synchronized maximum online-support peak supports support-level high-resistance checks.

The four peak indicators, their typical-cycle differences and their engineering-use boundaries are shown in Figure 4.

### 2.3. Statistical Roof-Weighting Labels and Spatial Indicators

Statistical roof-weighting labels were constructed separately from the asynchronous regional peak, synchronized face-mean peak, synchronized support-number-weighted face-mean peak, synchronized maximum section-mean peak and synchronized maximum support peak. These labels are pressure-threshold labels derived from hydraulic-support data; they are not independent roof-movement or roof-fracture labels.

The main thresholding rule was mean plus one standard deviation within the three-month pooled cycle samples. The old fixed threshold was 88.292 MPa. The pooled statistical threshold of ($P^{\mathrm{reg}}$) was 89.776 MPa, and that of ($P^{\mathrm{face}}$) was 65.614 MPa. Jaccard agreement was used to evaluate overlap between different statistical roof-weighting label sets.

For spatial interpretation, the dominant monitoring section was recorded for each cycle peak. The dominant section indicates whether the pressure response associated with the cycle peak mainly came from the conveyor side, middle or return-airway side. The three-section peak range was calculated to quantify along-face spatial non-uniformity.

### 2.4. Sampling-Interval and Phase-Sensitivity Experiments

Using the synchronized exact 3 min grid as the field reference, 15, 30 and 60 min down-sampled sequences were constructed. To avoid attributing the results to a single arbitrary sampling origin, all feasible phase offsets were traversed. For each target interval and phase offset, retained grid points were selected according to Equation (6):

(6) g mod (Δ/3)=ϕ

where *g* is the index of the synchronized grid, Δ is the target sampling interval and ϕ is the phase offset.

Thus, the target intervals corresponded to 5, 10 and 20 synchronized-grid steps, and the phase sweep included 5, 10 and 20 phase offsets, respectively. For each phase, the peak was recalculated within the same cycle boundaries. Evaluation metrics included cycle-peak underestimation, statistical roof-weighting event retention, dominant-section agreement and section-peak-range error. Underestimation was computed relative to the field high-frequency reference, and event retention was the fraction of synchronized face-mean statistical roof-weighting cycles preserved after down-sampling.

### 2.5. Uncertainty Estimation and Prediction-Error Diagnostic

Confidence intervals for differences, agreement rates and sampling effects were estimated using a moving-block bootstrap along the cycle index. A six-cycle block was selected to cover short-term serial dependence in the cycle-level peak-difference series; its autocorrelation decreased from 0.275 at lag 1 to 0.023 at lag 4. As a sensitivity check, block lengths of 1, 6 and 12 cycles produced similar 95% confidence intervals for the mean peak difference: 18.911–20.589, 18.651–20.755 and 18.538–20.856 MPa, respectively. For label-overlap metrics, bootstrap confidence intervals were calculated from repeated resampling of cycle indices. For sampling experiments, phase-level results were summarized together with cycle-level uncertainty.

A supplementary prediction diagnostic was conducted to clarify the boundary of point-prediction metrics. The target was the next-cycle synchronized face-mean peak. Inputs were recent completed-cycle peak features at the evaluated sampling interval. A ridge-regression model was tested under rolling temporal splits, with the ridge penalty selected inside the training period. This diagnostic was not treated as a model contribution; it was used only to test whether next-cycle mean absolute error (MAE) could replace direct evaluation of peak measurement and event retention.

### 2.6. Production Ledgers and Roadway-Stress Data for Joint-Event Screening

To test whether production constraints can help interpret a subset of statistical roof-weighting events, filling amount per unit advance was calculated for each cycle. Cycles below the three-month median of this quantity were defined as cycles with a relatively low filling amount per unit advance. Consecutive counts of such cycles, cumulative windows and filling-amount deficits per unit advance were used to describe short-term backfill history.

Roadway coal-stress response was summarized by the top-five mean stress-disturbance metrics within 0 h, ±3 h and ±6 h windows around the cycle start. A high stress-disturbance label was defined by the 75th percentile of the corresponding window metric. The joint event was defined by Equation (7):

(7) $$E_{c,w}^{\mathrm{joint}}=I(P_{c}^{\mathrm{face}}\geq T_{P})\cdot I(S_{c,w}\geq T_{S,w})$$

where (*T_P_*) is the synchronized face-mean statistical roof-weighting threshold and ($T_{S,w}$) is the stress-disturbance threshold. The joint event means that a statistical roof-weighting cycle was accompanied by a high roadway-stress disturbance. It is not an independent roof-fracture label. A 0–8 cycle distributed-lag analysis, matched-control comparison, threshold-grid robustness test and negative-control tests were further conducted. Because the lag analysis contained nine candidate tests, the results were interpreted as exploratory screening evidence and the multiple-comparison risk was reported. Here, S_c,w denotes the roadway-stress disturbance metric for cycle c within stress window w. Equations (1)–(7) formalize analytical procedures developed in this study. Equations (1)–(5) define pressure-peak aggregation, Equation (6) defines phase-resolved grid selection, and Equation (7) defines the joint pressure-stress event; hence, no single external equation source applies. Their engineering context is consistent with hydraulic-support and backfilling pressure-monitoring studies [2,24,25].

## 3. Results

### 3.1. Historical Peak-Source Reconstruction

**Figure 5:**
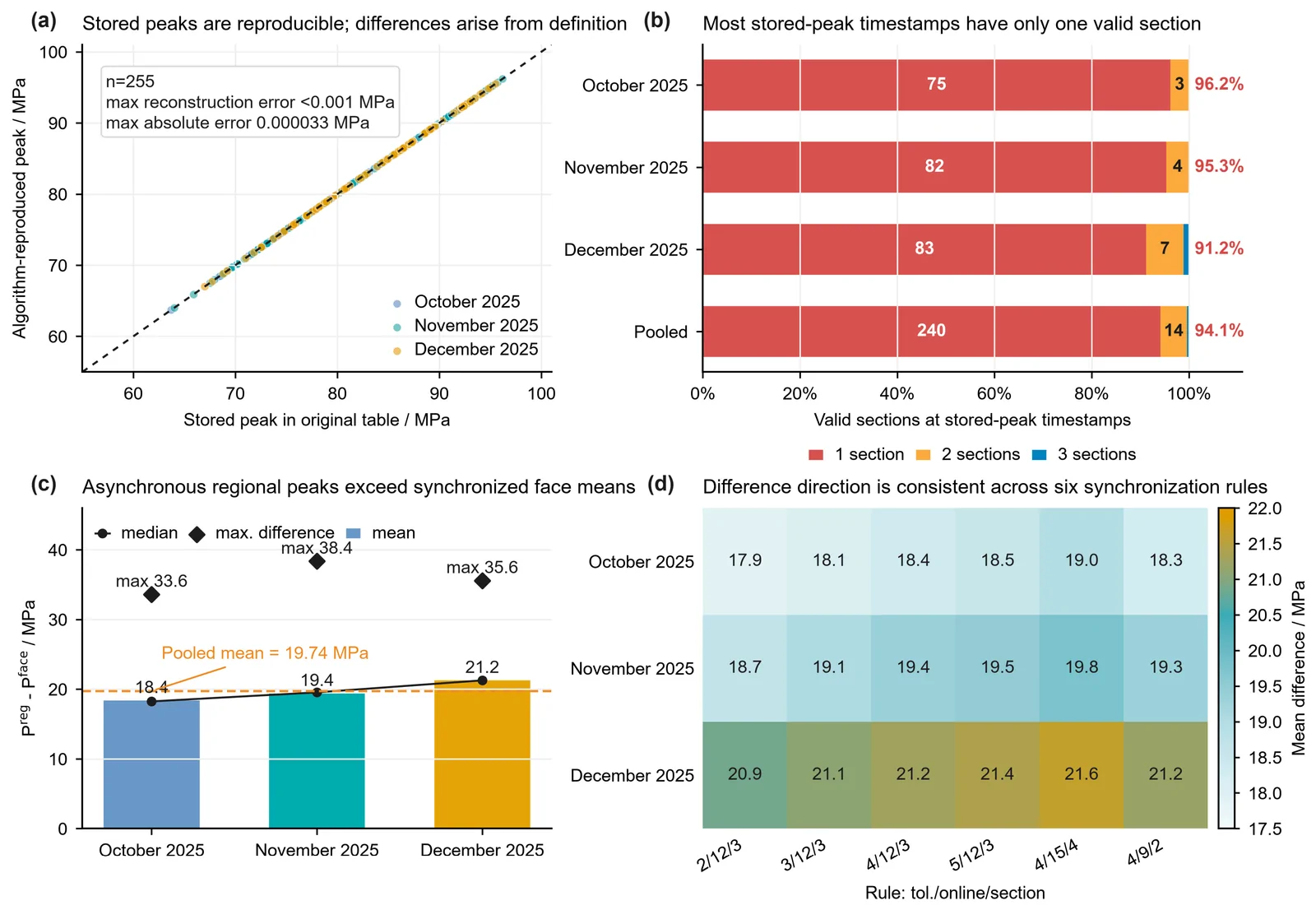
Historical aggregated peak-source audit. (**a**) Reproduction of the historical aggregated peak from the raw aggregation rule. (**b**) Number of valid monitoring sections at the peak timestamp. (**c**) Monthly difference between the asynchronous regional peak and synchronized face-mean peak from October to December 2025. (**d**) Robustness under six synchronization settings. The differences reflect different peak objects rather than errors in the raw pressure records. The synchronized comparison used 255 cycles satisfying the exact 3 min grid, 4 min matching tolerance, minimum 12 online supports and minimum 3 valid supports per section; no statistical roof-weighting threshold is applied in this source audit.

The historical aggregated peak field could be reconstructed from the raw pressure records, indicating that the subsequent differences were caused by peak-definition rules rather than file parsing. The peak-source audit and synchronization sensitivity are shown in Figure 5, and the monthly difference between the asynchronous regional peak and the synchronized face-mean peak is summarized in Table 2.

Among the 255 within-month high-frequency cycles, 240 historical peaks were determined at timestamps with only one valid monitoring section, 14 were determined by two sections and only one was determined by three sections. The asynchronous regional peak was therefore dominated by locally available section responses. After pooling the three months, it exceeded the synchronized face-mean peak by 19.736 MPa on average (95% confidence interval (CI): 18.608–20.591 MPa). The same direction was observed in all six synchronization-sensitivity settings, showing that the difference did not depend on a single tolerance or online-support requirement.

### 3.2. Peak Definition Changes Statistical Roof-Weighting Event Sets

**Figure 6:**
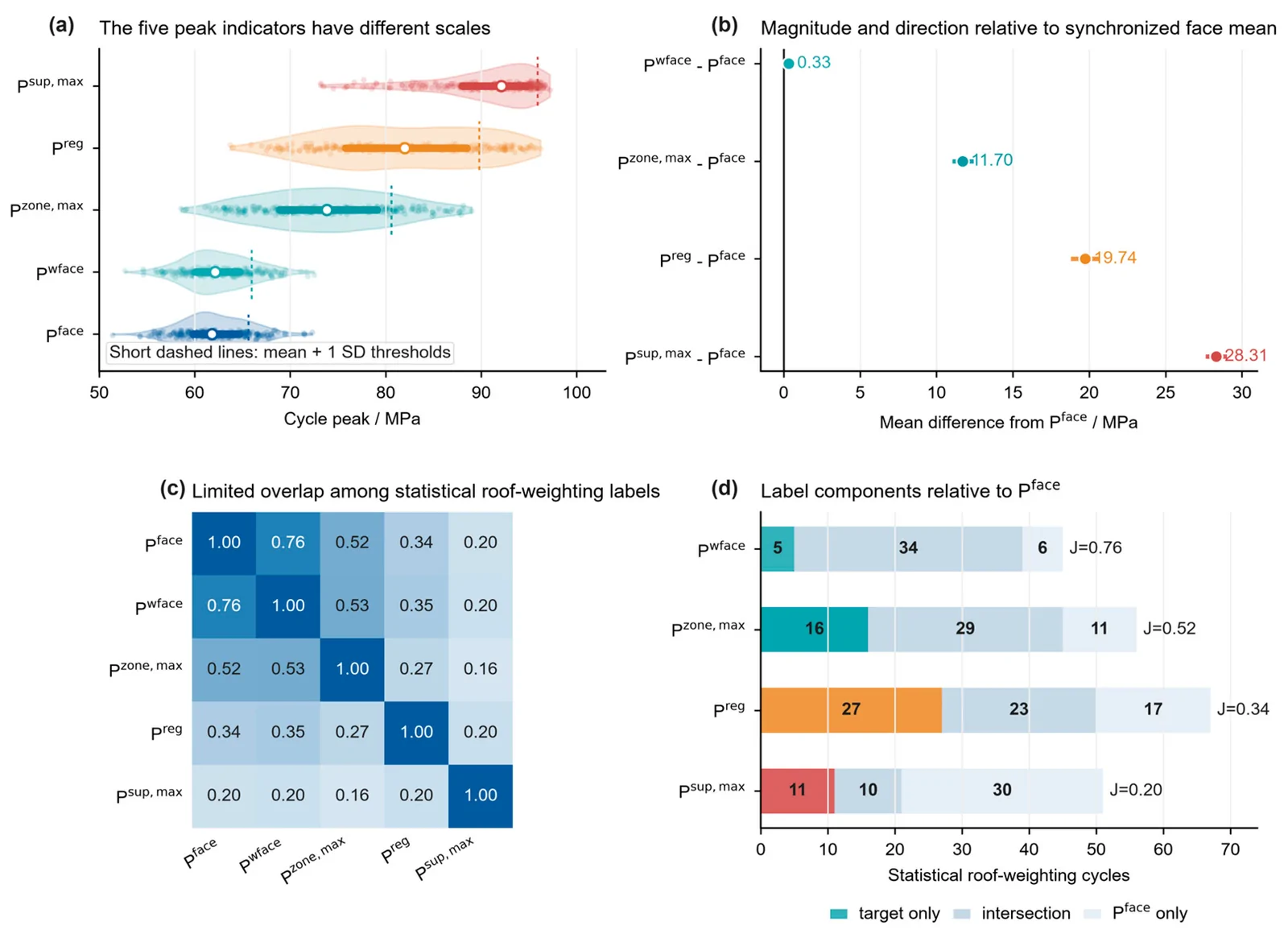
Distribution, difference and statistical roof-weighting label overlap of multiple peak indicators. (**a**) Cycle-level distributions of $P^{\mathrm{reg}}$, $P^{\mathrm{face}}$, $P^{\mathrm{wface}}$, $P^{\mathrm{zone},\max}$ and $P^{\sup,\max}$. (**b**) Difference of each indicator relative to $P^{\mathrm{face}}$. (**c**) Jaccard overlap among statistical roof-weighting labels. (**d**) Indicator-only, intersection and $P^{\mathrm{face}}$-only counts relative to the synchronized face-mean label. All labels are pressure-threshold labels, not independent roof-fracture labels. SD denotes standard deviation. In panels (**a**,**b**), dark blue denotes the synchronized face-mean peak, blue-green denotes the synchronized maximum section-mean peak, orange denotes the stored historical regional peak, red denotes the synchronized maximum online-support peak, and the lighter blue-green series denotes the support-number-weighted face mean. In panel (**d**), colours distinguish target-only, intersection and synchronized-face-mean-only components. Indicator-specific mean +1 SD thresholds were calculated from the 255-cycle sample. Synchronized indicators used the exact 3 min grid, 4 min matching tolerance, minimum 12 online supports and minimum 3 valid supports per section.

Different peak indicators produced different distributions, different offsets relative to ($P^{\mathrm{face}}$), and different statistical roof-weighting labels, as shown in Figure 6. The label-overlap and dominant-section results are summarized in Table 3.

Under the old fixed threshold, the asynchronous regional label identified 67 statistical roof-weighting cycles, whereas the synchronized face-mean label identified 40 cycles. The intersection contained 29 cycles, yielding a Jaccard agreement of 0.372 (95% CI: 0.231–0.490). When the asynchronous regional peak and synchronized face-mean peak were each assigned their own pooled statistical threshold, the asynchronous label identified 50 cycles, the synchronized label identified 40 cycles, and their intersection contained 23 cycles (Jaccard = 0.343). These results indicate that peak definition changes the statistical event set used for subsequent interpretation.

### 3.3. Synchronized Local Peaks Complement the Face-Mean Peak

**Figure 7:**
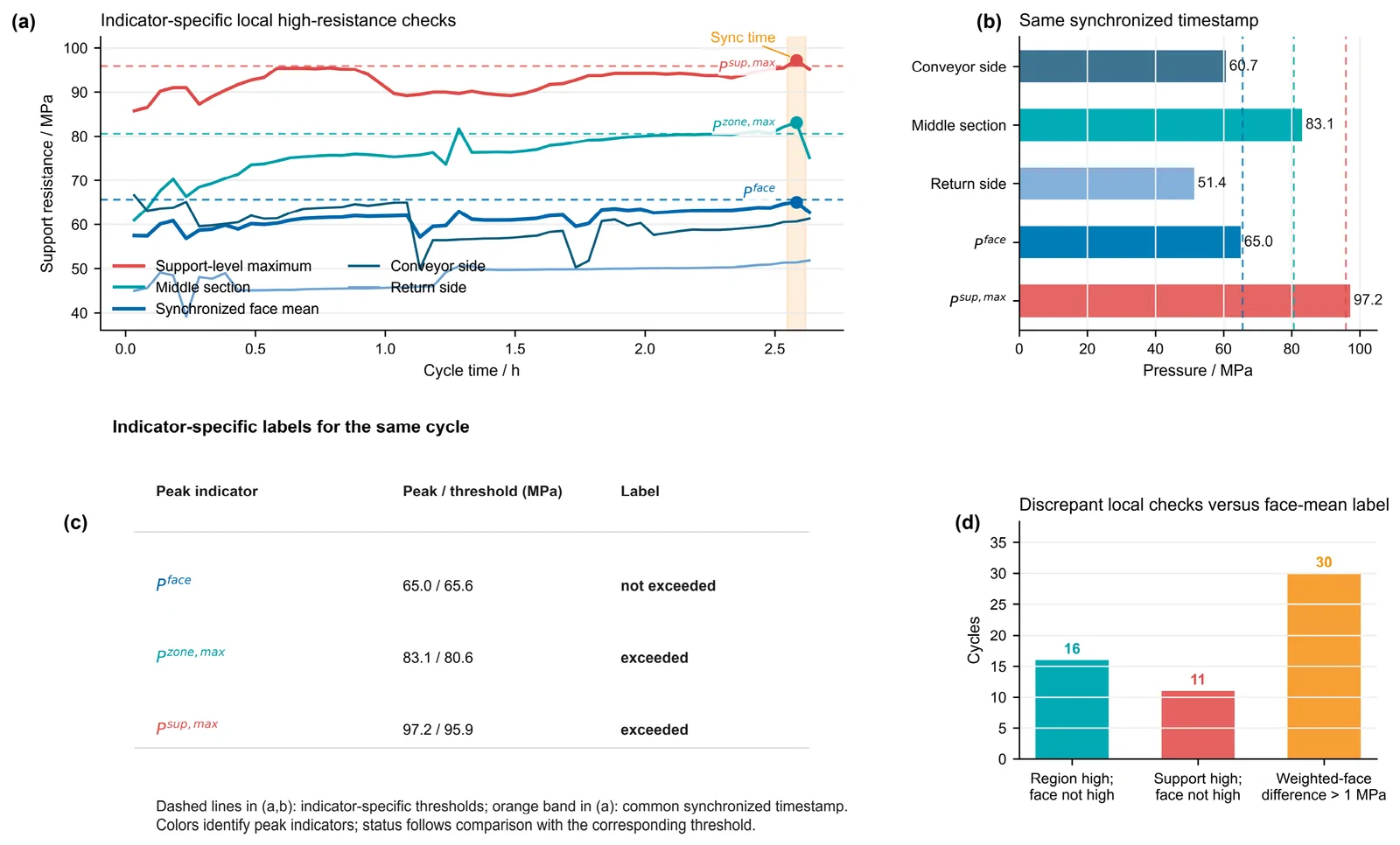
Local high-resistance responses under alternative peak definitions. (**a**) A typical cycle in which the middle-section and support-level local peaks may be attenuated by spatial averaging in the face mean. (**b**) Cross-section pressure values at the peak timestamp. (**c**) Different threshold judgements for $P^{\mathrm{face}}$, $P^{\mathrm{zone},\max}$ and $P^{\sup,\max}$. (**d**) Diagnostic counts for cycles where local synchronized peaks reached their thresholds but $P^{\mathrm{face}}$ did not, together with the weighted-mean sensitivity check. Thresholds are indicator-specific mean + 1 SD values calculated from the 255-cycle sample. The face-mean peak describes the average pressure response of the three monitored sections, the maximum section-mean peak supports regional high-resistance checks, and the maximum online-support peak supports support-level high-resistance checks. The analysis used the exact 3 min synchronized grid, a 4 min matching tolerance, at least 12 online supports and at least 3 valid supports per section.

The synchronized face-mean peak is useful for describing the average pressure response of the three monitored sections, but spatial averaging may attenuate local regional or support-level high-resistance responses. The local checks were based on the synchronized peak variables defined in Equations (2)–(5). A typical case and the corresponding diagnostic counts are shown in Figure 7. Table 4 summarizes the descriptive statistics and engineering-use boundary of each peak variable, and Table 5 summarizes the local-peak diagnostic results.

The synchronized maximum section-mean peak had a mean value of 73.831 MPa, 11.704 MPa higher than the synchronized face-mean peak. The synchronized maximum support peak had a mean value of 90.441 MPa, 28.314 MPa higher than the synchronized face-mean peak. Sixteen cycles reached the synchronized section-level statistical threshold without reaching the synchronized face-mean threshold, and 11 cycles reached the synchronized support-level threshold without reaching the face-mean threshold. Thus, using only $P^{\mathrm{face}}$ can under-represent local high-resistance responses. The historical asynchronous peak was closer to $P^{\mathrm{zone},\max}$ than to $P^{\mathrm{face}}$ in 253 of 255 cycles, indicating that the historical variable still has practical value for regional high-resistance screening if it is reported with the correct definition.

### 3.4. Peak Definition Changes Dominant-Section Interpretation

**Figure 8:**
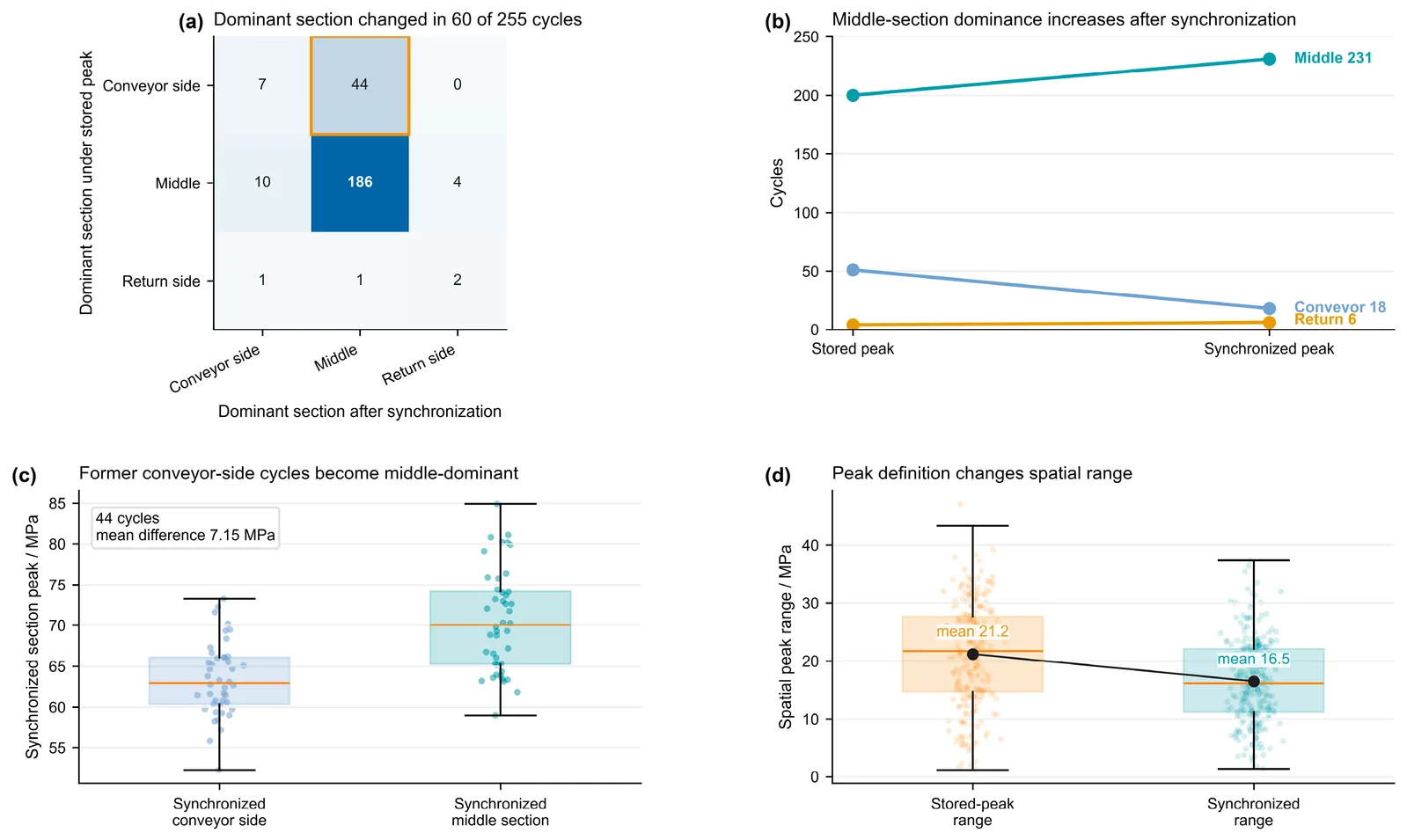
Dominant-section reclassification and spatial peak-range changes after synchronization. (**a**) Confusion matrix between the dominant section identified from the stored asynchronous peak and that identified from the synchronized peak. Cell shading represents the number of cycles. The orange frame highlights 44 cycles classified as conveyor-side dominant under the stored definition but middle-section dominant after synchronization. (**b**) Changes in the number of cycles assigned to each dominant section. The light-blue, teal and gold lines denote the conveyor side, middle section and return side, respectively. (**c**) Synchronized section-peak distributions for the 44 reclassified cycles. Dots represent individual cycles, boxes indicate the interquartile range, horizontal lines inside the boxes indicate medians, and whiskers extend to 1.5 times the interquartile range. (**d**) Spatial peak-range distributions under the stored asynchronous and synchronized definitions. Orange and teal boxes denote the stored-peak and synchronized ranges, respectively; black points indicate means and the black line connects the two means.

The dominant pressure-response section was consistent between the historical asynchronous peak and the synchronized face-mean peak in 195 of 255 cycles, corresponding to an agreement rate of 0.765 (95% CI: 0.710–0.804). In 60 cycles, the dominant-section judgement changed. Among the 51 cycles classified as conveyor-side dominant by the historical definition, 44 were reclassified as middle-section dominant under the synchronized definition. The reclassification pattern and spatial peak-range change are shown in Figure 8.

This result shows that asynchronous aggregation changes not only the magnitude of the cycle peak and the statistical roof-weighting label but also the inferred spatial source of the pressure response. A monitoring system that reports only an aggregated cycle peak without valid-section information may therefore obscure whether a peak came from a local regional response or from a synchronized mean pressure response of the three monitored sections.

### 3.5. Sampling Resolution Affects Peak Values, Event Retention and Spatial Range

**Figure 9:**
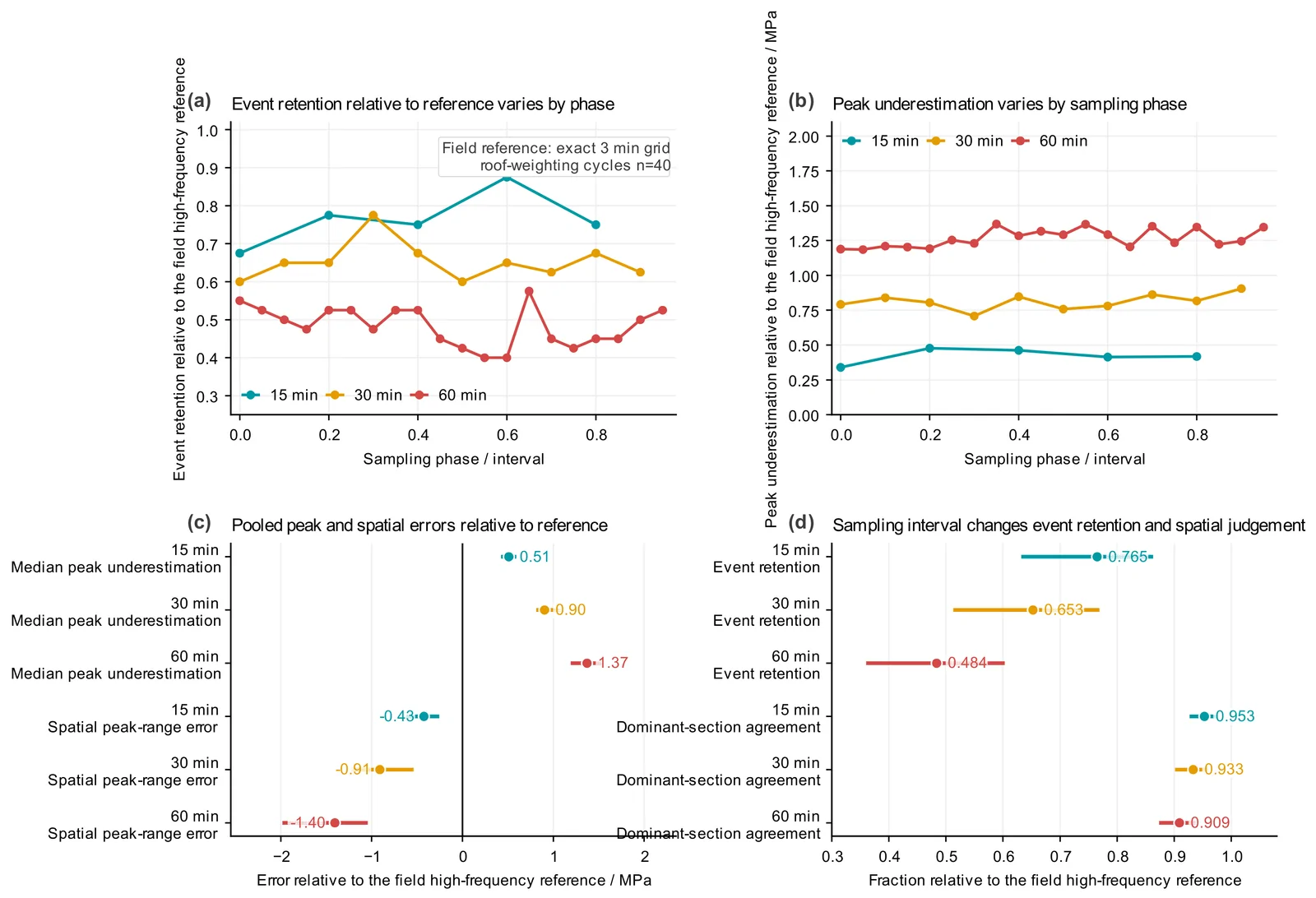
Sampling resolution and phase sensitivity. (**a**) Statistical roof-weighting event retention across sampling phases under 15, 30 and 60 min down-sampling. (**b**) Median cycle-peak underestimation relative to the field high-frequency reference across phases. (**c**) Cross-phase estimates of peak underestimation and section peak-range error. (**d**) Cross-phase estimates of event retention and dominant-section agreement. All losses are relative to the field high-frequency reference. The reference consists of 40 statistical roof-weighting cycles on an exact 3 min synchronization grid; the raw median interval was approximately 2.92 min. Target intervals were 15, 30 and 60 min, with all feasible phase offsets evaluated. Results apply to sampling-resolution assessment and description of the average pressure response of the three monitored sections under the stated inclusion criteria. The reference sequence satisfied a 4 min matching tolerance, minimum 12 online supports and minimum 3 valid supports per section; the analysis evaluates the face-mean indicator and is not a support-level safety check.

The phase sensitivity of statistical roof-weighting event retention, peak underestimation and section peak-range error under 15, 30 and 60 min down-sampling is shown in Figure 9. The cross-phase statistics are summarized in Table 6.

Relative to the synchronized field high-frequency reference, 15, 30 and 60 min down-sampling all reduced cycle peaks and statistical roof-weighting event retention. The median peak underestimation increased from 0.510 MPa at 15 min to 0.903 MPa at 30 min and 1.371 MPa at 60 min. Event retention decreased more sharply: the cross-phase average retention was 0.765, 0.653 and 0.484 at 15, 30 and 60 min, respectively. Sampling interval also affected spatial interpretation. The median section peak-range error was −0.425, −0.910 and −1.405 MPa, indicating that low-frequency instantaneous sampling compressed along-face spatial differences.

### 3.6. Point-Prediction Error Does Not Replace Monitoring-Reliability Evaluation

**Figure 10:**
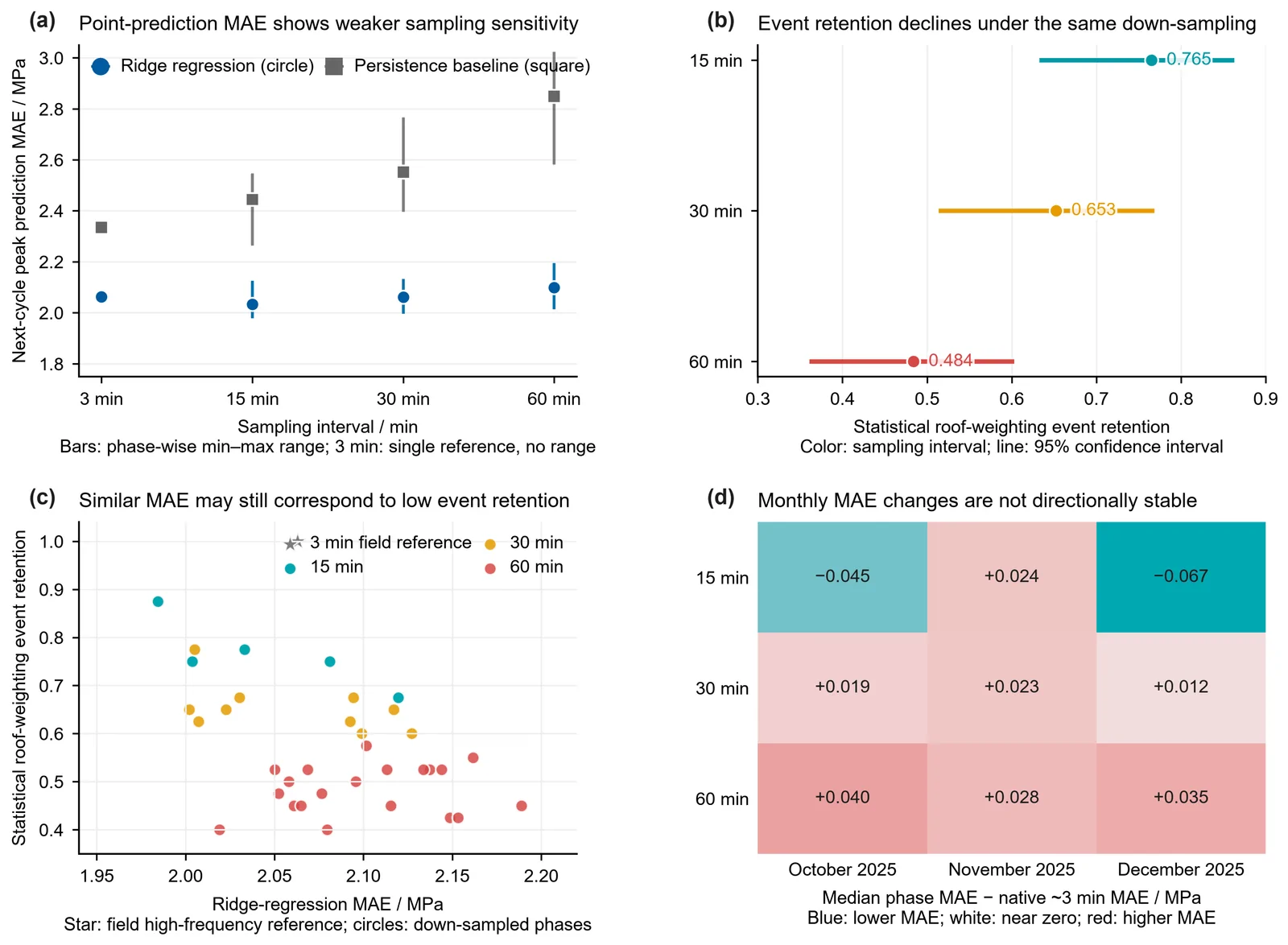
Boundary between point-prediction error and monitoring reliability. (**a**) Ridge-regression and persistence-baseline MAE for next-cycle synchronized face-mean peak prediction changed little across sampling intervals. (**b**) Event retention decreased under the same down-sampling experiment. (**c**) Similar MAE values can correspond to different event-retention levels. (**d**) Monthly MAE changes were not directionally stable. This diagnostic does not treat ridge regression as a methodological contribution. The prediction target is the synchronized face-mean indicator calculated under the same 4 min matching tolerance and support-completeness criteria; it represents the monitored-section mean response rather than a support-level safety metric.

The supplementary ridge-regression diagnostic compared next-cycle synchronized face-mean peak prediction errors under different sampling intervals. As shown in Figure 10, the point-prediction MAE changed only slightly, whereas statistical roof-weighting event retention clearly decreased.

With the field high-frequency reference input, the ridge-regression MAE for next-cycle synchronized face-mean peak prediction was 2.063 MPa. The phase-median MAE values at 15, 30 and 60 min were 2.033, 2.061 and 2.099 MPa, respectively. The 60 min input increased MAE by only 0.036 MPa relative to the field reference. This result does not indicate that sampling resolution is unimportant; it shows that next-cycle point-prediction error can remain stable while event retention and spatial peak-range estimation deteriorate. Monitoring-system evaluation should therefore include peak capture, event retention and spatial-response metrics in addition to prediction error.

### 3.7. Supplementary Analysis: Backfill-Advance and Roadway-Stress Joint Events

**Figure 11:**
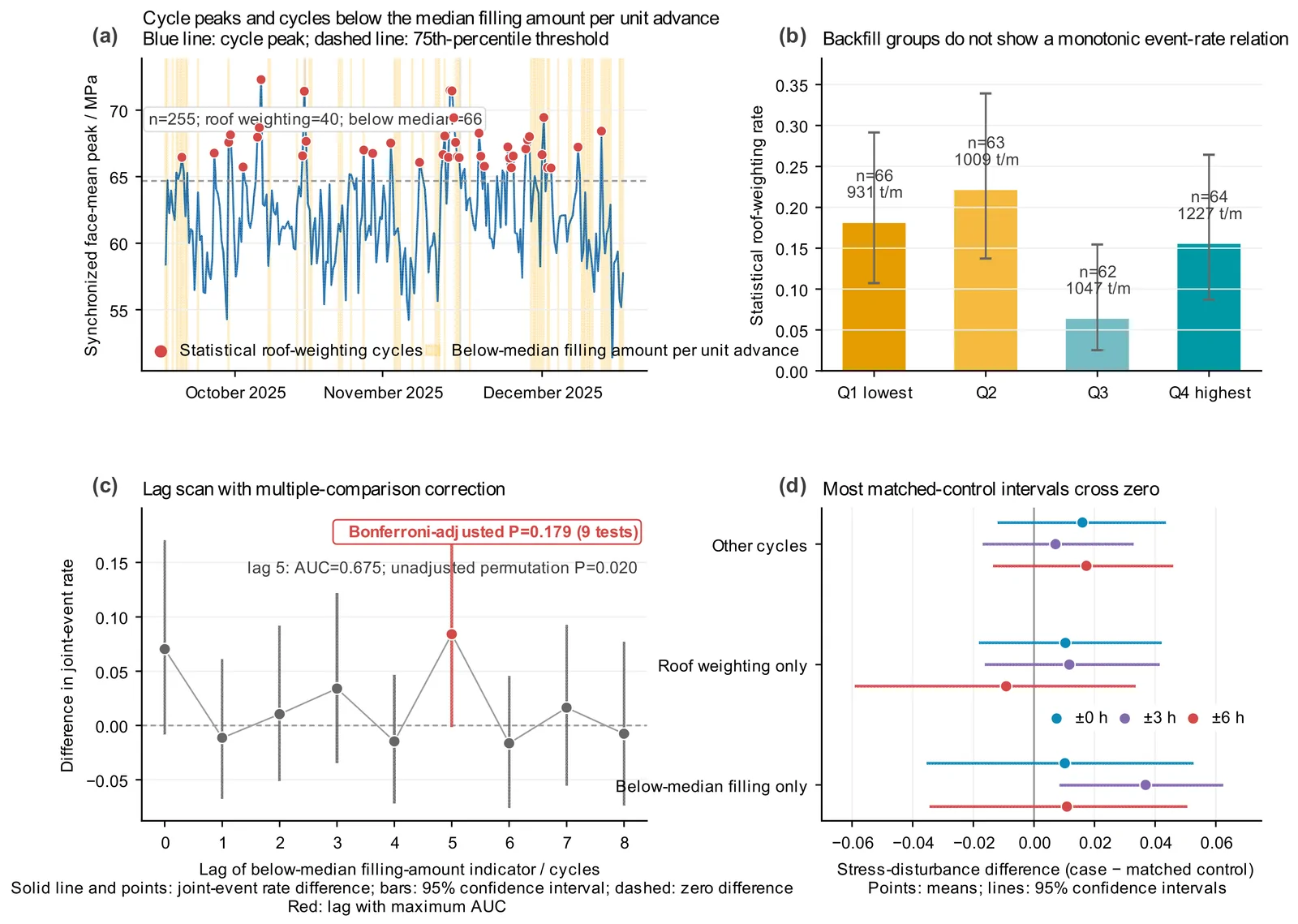
Backfill-advance and roadway-stress context for statistical roof-weighting event interpretation. (**a**) Cycle-scale synchronized face-mean peak, statistical roof-weighting cycles and windows comprising cycles with a relatively low filling amount per unit advance. (**b**) Statistical roof-weighting rate by quartile of unit-advance filling amount. (**c**) Lag scan of the association between lower filling amount per unit advance and the joint event defined as synchronized statistical roof weighting with high 0 h roadway-stress disturbance. (**d**) Roadway-stress disturbance difference after matched-control comparison. The figure provides event-screening context and is not an independent roof-fracture validation. The filling-amount stratifier was defined as a cycle with filling amount per unit advance below the three-month median. This production-ledger variable was used to examine its association with event occurrence and does not independently label roof fracture. Statistical roof-weighting labels were derived from the synchronized face-mean indicator using its mean + 1 SD threshold over 255 cycles and the stated synchronization-completeness criteria.

The joint analysis of backfill-advance ledgers and roadway-stress data is shown in Figure 11.

When the outcome was the ordinary synchronized face-mean statistical roof-weighting label, the strongest lag-5 association with a relatively low filling amount per unit advance was weak (area under the receiver operating characteristic curve (AUC) = 0.543, permutation *p* = 0.282). When the outcome was narrowed to the joint event of synchronized statistical roof weighting with high 0 h roadway-stress disturbance, the lag-5 lower-filling-amount exposure showed an event-rate difference of +0.084, an odds ratio of 4.304, AUC = 0.675 and permutation *p* = 0.020. A six-cycle cumulative count of cycles with a relatively low filling amount per unit advance reached AUC = 0.709 with permutation *p* = 0.030.

These results are exploratory. The 0–8 cycle lag scan involved nine candidate tests; under Bonferroni correction, the 0.05 significance threshold is approximately 0.0056, and the lag-5 permutation *p* value becomes 0.179 after correction. The joint event count was only 10, so AUC = 0.675 can be affected by individual cycles. In the threshold-grid robustness test, only 0.048 of 250 eligible parameter combinations satisfied the predefined lag-5 screening criterion, indicating a narrow support region. The complete diagnostic is reported in Supplementary Table S1. The supplementary analysis brings the solid backfilling production context into event screening, but it does not support interpreting cycles with a relatively low filling amount per unit advance as the cause of roof weighting or as roof-fracture evidence.

## 4. Discussion

### 4.1. Peak Indicators Should Match Monitoring Objectives

The synchronized face-mean peak and the asynchronous regional peak answer different engineering questions. The asynchronous regional peak can reproduce the historical aggregation algorithm and indicate regional high-resistance responses, but its peak timestamp often contains only one valid section. It should therefore not be named or used as a synchronized average peak of the three monitored sections. The synchronized face-mean peak is suitable for describing the average pressure response of the three monitored sections and for cross-cycle statistical comparison, but spatial averaging may attenuate short-duration regional or support-level high-resistance responses.

For this reason, local high-resistance checks should report $P^{\mathrm{zone},\max}$ or $P^{\sup,\max}$ together with $P^{\mathrm{face}}$. In this study, 16 cycles reached the synchronized section-level threshold without reaching the synchronized face-mean threshold, and 11 cycles reached the synchronized support-level threshold without reaching the synchronized face-mean threshold. The historical asynchronous peak was closer to the synchronized maximum section-mean peak in 253 of 255 cycles, confirming that it remains informative as a regional response indicator when its definition is stated correctly.

Multi-support mining-pressure monitoring reports should specify at least four items: the peak object being calculated, the data completeness at the peak timestamp, the threshold source used for statistical roof-weighting labels, and the engineering use of the indicator. If these items are omitted, a subsequent prediction model may compare peak variables with different meanings while still producing seemingly small numerical errors.

From a pressure-sensor-system perspective, cycle peaks should not be treated as post-processing variables independent of the acquisition chain. Raw timestamps, support IDs, section IDs, online status, synchronization tolerance, valid-sensor count and window-maximum source should be retained. Safety-oriented reports should output regional and support-level peaks together with the face-mean peak so that average pressure response of the three monitored sections and local high-resistance response can be interpreted in one monitoring framework.

### 4.2. Sampling Strategy Should Follow Event-Retention Objectives

Relative to the field high-frequency reference, 15–60 min instantaneous down-sampling systematically reduced cycle peaks and statistical roof-weighting event retention. This study does not propose a universal sampling standard. A reasonable sampling interval should depend on the acceptable event-miss rate, peak duration, regional or monitored-section mean-pressure objective, storage of window maxima and field communication capacity.

If communication or storage restrictions require a longer upload interval, two types of data should be distinguished. Low-frequency instantaneous values can support trend statistics and long-term operation summaries. Window maxima or event-triggered records may therefore provide a more appropriate strategy when preservation of short-duration roof-weighting events and local high-resistance responses is required. The results also show that longer sampling intervals compress section peak range; therefore, low-frequency instantaneous sequences should not be used alone to infer spatial uniformity or regional load concentration.

### 4.3. Production Ledgers Are Useful for Joint-Event Screening

The backfill-advance ledger did not improve ordinary next-cycle point prediction and did not stably screen ordinary synchronized statistical roof-weighting events. It showed an exploratory signal only when the event definition was restricted to synchronized statistical roof weighting accompanied by a high 0 h roadway-stress disturbance. This suggests that production ledgers may be more useful as event-stratification variables than as direct predictors in this dataset.

These findings motivate a hypothesis-driven two-stage workflow for future validation. The first stage ensures that pressure-peak definition, synchronization rule and sampling interval can stably preserve statistical roof-weighting events. The second stage uses backfill-advance ledgers and independent stress responses to stratify statistical roof-weighting cycles into ordinary pressure-threshold events and joint events that warrant additional field review. Such joint events should prompt checks of filling records, support state and roadway-stress changes, but they should not be treated as independently verified roof-fracture events without additional physical measurements.

### 4.4. Limitations

This study is based on one large-height solid backfilling working face and three months of high-frequency monitoring data. The numerical thresholds and retention rates strictly apply to similar high-frequency asynchronous monitoring conditions in high-filling-ratio backfilling faces. For caving faces, hard-roof conditions or other monitoring systems, the audit framework can be reused, but thresholds and numerical conclusions must be recalibrated. Analysing other months may change pressure distributions, statistical thresholds and event counts as production and geological conditions evolve; using other sampling intervals may likewise change phase-specific retention according to interval length and peak duration. The analytical framework remains applicable, but the numerical results must be recalculated for the new period and interval.

The synchronized 3 min-grid sequence serves as the field high-frequency reference, not a true continuous-time peak record. Therefore, the reported 15–60 min down-sampling losses are relative losses with respect to the field reference rather than absolute losses relative to continuous pressure peaks. Sensor calibration drift, communication-packet loss and raw recorder window policies were not fully available, so hardware error, communication error and post-processing aggregation error cannot be completely separated.

Backfill amount and advance distance were obtained from ledgers and allocated to cycles, so they are proxy variables rather than real-time filling ratio, equivalent mining height or overburden movement space. Roadway coal-stress data provide an independent stress-response check but do not prove roof fracture. The study audits monitoring definitions, event retention and joint-event screening; it does not claim to identify roof-fracture states or overburden movement modes. Future work should add roof displacement, separation, microseismic records, safety-valve action logs or multi-face independent validation.

## 5. Conclusions

1. The stored cycle peak field in the existing dataset is an asynchronous available-section mean maximum. Among 255 cycles, 240 historical peaks were determined at timestamps with only one valid section, indicating that unsynchronized multi-support records can shift the resulting peak toward locally dominated section-level responses.

2. The historical asynchronous regional peak exceeded the synchronized face-mean peak by 19.736 MPa on average. The old fixed-threshold asynchronous statistical roof-weighting labels and synchronized face-mean labels had a Jaccard agreement of only 0.372, showing that peak-definition choice changes the event set.

3. The synchronized face-mean peak is suitable for describing the average pressure response of the three monitored sections but may attenuate local responses through spatial averaging. Sixteen cycles reached the synchronized maximum section-mean threshold without reaching the face-mean threshold, and 11 cycles reached the synchronized maximum support threshold without reaching the face-mean threshold.

4. The historical asynchronous peak was closer to the synchronized maximum section-mean peak in 253 of 255 cycles. Regional high-resistance checks should therefore report $P^{\mathrm{zone},\max}$, and support-level checks should report $P^{\sup,\max}$, rather than relying only on $P^{\mathrm{face}}$.

5. Relative to the field high-frequency reference, 15, 30 and 60 min down-sampling reduced cycle peaks and statistical roof-weighting event retention. At 60 min, the cross-phase average event retention decreased to 0.484.

6. Longer sampling intervals also compressed section peak range. When window maxima or event-triggered records are not stored, low-frequency instantaneous sequences should not be used alone for short-duration roof-weighting event checks or regional load-concentration interpretation.

7. Backfill-advance ledgers did not improve ordinary synchronized peak prediction and did not stably screen ordinary synchronized statistical roof-weighting events. Their lag-5 signal for the joint event of synchronized statistical roof weighting with high 0 h stress disturbance should be interpreted only as exploratory association after multiple-comparison correction.

8. Mining-pressure sensor reports should explicitly state peak definition, synchronization rule, valid-section number, sampling interval, statistical roof-weighting threshold and window-maximum preservation. The average pressure response of the three monitored sections, regional high-resistance checks, support-level high-resistance checks and joint-event interpretation should be reported as related but separate monitoring outputs.

## Figures and Tables

**Table 1:** High-frequency monitoring data and production-cycle samples from the Mine 01 working face.

Month	Valid Pressure Records	Monitored Supports	Sections	Within-Month Cycles	Median Raw Interval/Min	Start Date	End Date
2025-10	174,905	15	3	78	2.92	2025-10-01	2025-10-31
2025-11	181,744	15	3	86	2.92	2025-11-01	2025-11-30
2025-12	232,362	15	3	91	2.92	2025-12-01	2025-12-31
Pooled	589,011	15	3	255	approx. 2.92	2025-10-01	2025-12-31

Note: The raw sampling interval was calculated from adjacent records of the same monitored support. The pooled value is a descriptive field-reference interval for the three-month dataset. The three sections refer to the conveyor-side, middle, and return-airway-side monitoring sections, respectively.

**Table 2:** Difference between the asynchronous regional peak and the synchronized face-mean peak.

Month	Cycles	Peaks Determined by One Section	Peaks Determined by Two Sections	Peaks Determined by Three Sections	One-Section Ratio	Mean Difference/MPa	Median Difference/MPa	Maximum Difference/MPa
October 2025	78	75	3	0	96.15%	18.367	18.243	33.606
November 2025	86	82	4	0	95.35%	19.378	19.538	38.354
December 2025	91	83	7	1	91.21%	21.249	21.260	35.566
Pooled	255	240	14	1	94.12%	19.736 (18.608–20.591)	19.623 (18.317–20.792)	38.354

Note: Values in parentheses are 95% confidence intervals estimated by a moving-block bootstrap with a six-cycle block length. The 1-, 6- and 12-cycle block-length sensitivity checks yielded overlapping confidence intervals for the pooled mean peak difference. The synchronized comparison contains 255 cycles satisfying the exact 3 min grid, 4 min tolerance and stated support-completeness criteria; This table quantifies indicator differences and is not a support-level safety assessment.

**Table 3:** Effect of peak definition on statistical roof-weighting labels and dominant-section judgement.

Comparison	Asynchronous/Old Count	Synchronized Count	Intersection or Agreement	Jaccard or Agreement Rate (95% CI)
Old fixed-threshold asynchronous label vs. synchronized statistical label	67	40	29	0.372 (0.231–0.490)
Asynchronous statistical label vs. synchronized statistical label	50	40	23	0.343 (0.204–0.466)
Asynchronous peak dominant section vs. synchronized peak dominant section	255	255	195 same, 60 changed	0.765 (0.710–0.804)

Note: Statistical roof-weighting labels are pressure-threshold labels. The dominant section represents the pressure-response source associated with the peak. Indicator-specific mean + 1 SD thresholds were calculated from 255 cycles. Synchronized indicators used the exact 3 min grid, 4 min tolerance and stated support-completeness criteria.

**Table 4:** Peak variables and their engineering-use boundaries.

Peak Variable	Engineering-Use Boundary	Mean/MPa	Statistical Threshold/MPa	Statistical Roof-Weighting Cycles	Cycles
$P^{\mathrm{reg}}$	Reproduces the historical algorithm; should not be interpreted alone as a synchronized local peak or three-section mean peak	81.863	89.776	50	255
$P^{\mathrm{face}}$	Synchronized equal-section face-mean load background; not for local support-safety judgement	62.127	65.614	40	255
$P^{\mathrm{wface}}$	Support-number-weighted synchronized face mean; sensitivity check for unequal section sizes	62.459	65.966	39	255
$P^{\mathrm{zone},\max}$	Synchronized maximum section-mean peak; regional high-resistance check	73.831	80.592	45	255
$P^{\sup,\max}$	Synchronized maximum online-support peak; support-level high-resistance check	90.441	95.903	21	255

Note: Except for $P^{\mathrm{reg}}$, all synchronized variables were calculated on the same time grid with a 4 min nearest-neighbour tolerance, at least 12 online supports and at least 3 valid supports per section. Indicator-specific thresholds were used only for comparing event sets.

**Table 5:** Local synchronized peak diagnostics relative to the synchronized face-mean peak.

Diagnostic Item	Cycles	Proportion	Interpretation
Stored regional peak closer to maximum section-mean peak than to face-mean peak	253	0.992	The historical asynchronous peak mainly behaves as a regional high-resistance indicator
Section-mean threshold exceeded; face-mean threshold not exceeded	16	0.063	Regional check differs from the face-mean label owing to spatial aggregation and indicator-specific thresholds
Online-support threshold exceeded; face-mean threshold not exceeded	11	0.043	Support-level check differs from the face-mean label owing to spatial aggregation and indicator-specific thresholds

Note: $P^{\mathrm{face}}$ represents the synchronized average pressure response of the three monitored sections. $P^{\mathrm{zone},\max}$ and $P^{\sup,\max}$ are required for regional and support-level high-resistance checks. As a weighted-mean sensitivity check, $|P^{\mathrm{wface}}-P^{\mathrm{face}}|>1$ MPa occurred in 30 cycles (0.118 of 255 cycles), indicating that unequal monitored-support numbers caused visible but secondary differences between equal-section and weighted face means. Thresholds in Table 5 are indicator-specific mean +1 SD values calculated from 255 cycles. The exact 3 min synchronized grid, 4 min matching tolerance, minimum 12 online supports and minimum 3 valid supports per section were used. The stored regional peak, face-mean peak, maximum section-mean peak and maximum online-support peak retain the engineering-use boundaries defined in Table 4: historical-variable reproduction, description of the average pressure response of the three monitored sections, regional checks and support-level checks, respectively.

**Table 6:** Peak underestimation, event retention and spatial range error under different sampling intervals.

Sampling Interval/min	Median Peak Underestimation/MPa	Statistical Roof-Weighting Event Retention	Dominant-Section Agreement	Median Section-Range Error/MPa	Cycles	Reference Roof-Weighting Cycles
15	0.510 (0.444–0.571)	0.765 (0.635–0.860)	0.953 (0.930–0.971)	−0.425 (−0.600–0.275)	255	40
30	0.903 (0.832–1.057)	0.653 (0.516–0.766)	0.933 (0.905–0.956)	−0.910 (−1.205–0.558)	255	40
60	1.371 (1.211–1.511)	0.484 (0.363–0.600)	0.909 (0.877–0.934)	−1.405 (−1.963–1.063)	255	40

Note: Values in parentheses are 95% confidence intervals. Positive underestimation means that the down-sampled peak is lower than the field high-frequency reference. Negative section-range error means that spatial differences are compressed. The reference face-mean sequence used the exact 3 min grid, 4 min matching tolerance, minimum 12 online supports and minimum 3 valid supports per section; This table concerns monitored-section mean response and spatial-range preservation, not single-support safety.

## Data Availability

De-identified data and scripts are provided in the Supplementary Package; raw records are confidential but available upon reasonable request from the corresponding author.

## Supplementary Materials

The following supporting information can be downloaded at: https://www.mdpi.com/article/10.3390/s26175660/s1, Table S1 reports the backfill-advance and roadway-stress joint-event screening diagnostics, including ordinary peak-prediction baselines, lag-scan statistics, threshold-grid robustness and negative controls. The table is derived from the synchronized peak audit, distributed-lag event study and stress-response validation scripts in the accompanying data-code package.
